# HHI: How housing intervention programmes are improving health outcomes for children in New Zealand

**DOI:** 10.23889/ijpds.v9i5.2893

**Published:** 2024-09-18

**Authors:** Ellie Johnson, Nevil Pierse, Elinor Chisholm

**Affiliations:** 1 University of Otago, Wellington

## Objectives

It is estimated that around 30,000 New Zealand (NZ) children are hospitalised each year for preventable conditions related to poor-quality housing. To combat the high levels of housing-related illness in NZ, the Healthy Homes Initiative (HHI) was established. The HHI is a government-funded, multicomponent housing intervention programme operating with the aim of reducing the incidence of housing-related illness in children. The objective of this study was to evaluate the impact of the HHI programme on health outcomes of participating families.

## Approach

The Integrated Data Infrastructure (IDI) is a database of linked microdata maintained by Statistics NZ. Deidentified HHI referral data were linked to the IDI, and we used government administrative data to explore the impact of the HHI on health outcomes.

## Results

The HHI dataset linked to the IDI provided referral information for over 21,000 participants of the programme. Characteristics of the cohort included a higher proportion of participants who identified as Māori or Pacific Islander and lower socio-economic status than the general NZ population. Odds ratios were calculated for all variables in the model and showed lower odds of hospitalisation associated with participation in the HHI programme.

## Conclusion

This study demonstrates how housing-intervention programmes can improve health outcomes for those at risk of illness related to housing. These programmes are imperative for addressing the burden of disease and significant health inequities driven by exposure to substandard housing in NZ. This study shows how data linkage methods and administrative data can be used to evaluate large-scale government housing programmes.

